# *α*-MoO_3_ Crystals with a Multilayer Stack Structure Obtained by Annealing from a Lamellar MoS_2_/g-C_3_N_4_ Nanohybrid

**DOI:** 10.3390/nano8070559

**Published:** 2018-07-22

**Authors:** Pablo Martín-Ramos, Ignacio A. Fernández-Coppel, Manuel Avella, Jesús Martín-Gil

**Affiliations:** 1 Department of Agricultural and Environmental Sciences, EPS, Instituto de Investigación en Ciencias Ambientales (IUCA), University of Zaragoza, Carretera de Cuarte s/n, 22071 Huesca, Spain; 2 Engineering of Manufacturing Processes group, School of Industrial Engineering, University of Valladolid, C/ Francisco Mendizábal 1, 47014 Valladolid, Spain; ignacio.alonso.fernandez-coppel@uva.es; 3 Unidad de Microscopía Avanzada, Parque Científico UVa, Universidad de Valladolid, Paseo Belén 11, 47011 Valladolid, Spain; um.parque.cientifico@uva.es; 4 Agriculture and Forestry Engineering Department, ETSIIAA, Universidad de Valladolid, Avenida de Madrid 44, 34004 Palencia, Spain; mgil@iaf.uva.es

**Keywords:** *α*-MoO_3_, carbon nitride, g-C_3_N_4_, molybdenum trioxide, nanoplates, synthesis

## Abstract

Transition metal oxides and chalcogenides have recently attracted great attention as the next generation of 2-D materials due to their unique electronic and optical properties. In this study, a new procedure for the obtaining of highly crystalline *α*-MoO_3_ is proposed as an alternative to vapor-phase synthesis. In this approach, a first reaction between molybdate, citrate and thiourea allowed to obtain MoS_2_, which—upon calcination at a temperature of 650 °C in the presence of g-C_3_N_4_—resulted in MoO_3_ with a definite plate-like shape. The colorless (or greenish) *α*-MoO_3_ nanoplates obtained with this procedure featured a multilayer stack structure, with a side-length of 1–2 μm and a thickness of several nanometers viewed along the [010] direction. The nucleation-growth of the crystal can be explained by a two-dimensional layer-by-layer mechanism favored by g-C_3_N_4_ lamellar template.

## 1. Introduction

MoO_3_ is a versatile compound with well-recognized applications in electronics, photo- and electrocatalysis, electrode materials for batteries and pseudocapacitors, gas sensing, superconductors, lubricants, thermoelectric and electrochromic systems, etc., as discussed in detail in the recent review paper by de Castro, et al. [1].

In particular, stoichiometric and intrinsic MoO_3_ in its *α*-phase is an *n*-type semiconductor with a wide bandgap energy of ca. 3 eV (a range from 2.7 to 3.2 eV has been reported), an electron affinity >6 eV and an ionization energy >9 eV [2,3]. Its high work function has led to extensive applications as an anode interfacial layer in electronics (e.g., in solar cells, light-emitting diodes, 2-D field-effect transistors and photodetectors) [2,3,4,5].

Orthorhombic *α*-MoO_3_ features a layered crystal structure, which offers the possibility to create 2-D morphologies. Those layers are made of atomically thin sheets featuring a thickness of ≈0.7 nm, composed of double layers of linked and distorted MoO_6_ octahedra. In the vertical [010] direction, the distorted MoO_6_ octahedra are held together by van der Waals’ forces, resulting in stratification, while the internal interactions in the octahedra are dominated by strong covalent and ionic bonds [6,7].

Sheet-like orthorhombic *α*-MoO_3_ nanostructures are usually prepared by a simple hydrothermal method using ammonium heptamolybdate tetrahydrate and nitric acid [8,9], although both liquid- and vapor-phase-based alternative approaches have been devised for synthesizing and depositing this oxide. Actually, sputtering is now the most commonly used technique for industrial scale deposition of well-defined, large-area crystalline films of molybdenum oxide [10,11]. 

Aforementioned approaches have some limitations: physical vapor deposition (PVD) and chemical vapor deposition (CVD) methods have substantial energy requirements, rely on complex equipment and need expert operation skills; on the other hand, most liquid-phase synthesis techniques have problems in terms of relative scalability and repeatability, as well as in terms of their ability to produce molybdenum oxides with high crystallinity, controlled stoichiometry, and morphology.

As most applications require clean and large-sized flakes, this pinpoints a clear need to keep exploring new ways to prepare high quality single-layer transition metal oxides and chalcogenides with high yield. In this work we describe a new procedure to obtain highly ordered multi-layer stacks of molybdenum trioxide (*α*-MoO_3_) from a molybdate, citrate and thiourea mixture in propylene carbonate solution upon heating at 650 °C, using carbon nitride (g-C_3_N_4_) as a lamellar template. The resulting material may find application, for instance, in the field of clean energy (provided that 2-D *α*-MoO_3_ nanosheets have recently been reported to be strong candidates for electrocatalytic hydrogen evolution reaction [12]) or in ultrasensitive plasmonic biosensing [13].

## 2. Materials and Methods

### 2.1. Reagents and Synthesis

Ammonium heptamolybdate tetrahydrate ((NH_4_)_6_Mo_7_O_24_·4H_2_O, CAS No. 12054-85-2, puriss, ≥99%), citric acid monohydrate (C_6_H_8_O_7_·H_2_O, CAS No. 5949-29-1, ACS reagent, ≥99.0%), thiourea (CH_4_N_2_S, CAS No. 62-56-6, ACS reagent, ≥99.0%) and propylene carbonate (C_4_H_6_O_3_, CAS No. 108-32-7, anhydrous, 99.7%) were purchased from Sigma-Aldrich Química SL (Madrid, Spain), and were used without further purification. g-C_3_N_4_ was prepared according to the procedure reported in [14]. 

#### 2.1.1. Synthesis of MoS_2_

Firstly, ammonium heptamolybdate tetrahydrate ((NH_4_)_6_Mo_7_O_24_∙4H_2_O) (4 mmol) was dissolved in 100 mL of distilled water under continuous stirring, and 2 g of citric acid monohydrate (C_6_H_8_O_7_·H_2_O) were then added to the solution, resulting in a pH of 4. Subsequently, 10 mmol of thiourea were added to the solution mixture and the dispersion was sonicated for 60 min (with a probe-type UIP1000hdT ultrasonicator; Hielscher, Teltow, Germany; 1000 W, 20 kHz) in 10 periods of 2 min each, keeping the temperature below 40 °C. The initial greenish black color changed to dark red and, after heating at 90 °C for 1 h with stirring on a heating magnetic stirrer, color changed from red to black. After centrifugation at 4000 rpm for 1 h, a precipitate was formed, which was washed 3 times with distilled water and ethanol and then dried at 60 °C for 24 h. 1 g of this precipitate was introduced into a 40 mL Teflon-lined stainless steel autoclave and 30 mL of propylene carbonate were added, followed by stirring and heating at 200 °C for 24 h, yielding a solution that would contain [Mo(CH_4_N_2_S)_2_O_3_]) (or, secondarily, some [(MoO_2_)_2_O(C_6_H_8_O_7_)_2_] as a transient species). By centrifugation of this solution, a precipitate was obtained, in which MoS_2_ would be the main component [15,16]. This precipitate was dried at 150 °C for 24 h. 

Two proposed reaction mechanisms would be:

(1) $$2\left[\mathrm{Mo}{\left({\mathrm{CH}}_{4}N_{2}S\right)}_{2}O_{3}\right]+8{\;O}_{2}\to {\mathrm{Mo}}_{2}S+3{\;\mathrm{SO}}_{2}↑+\;4{\;\mathrm{CO}}_{2}↑+\;8{\;H}_{2}O↑+\;2{\;N}_{2}↑$$

(2) $$2\left[{\left({\mathrm{MoO}}_{2}\right)}_{2}O{\left(C_{6}H_{8}O_{7}\right)}_{2}\right]+2{\;\mathrm{CH}}_{4}N_{2}S+17{\;O}_{2}\to 2{\;\mathrm{Mo}}_{2}S+26{\;\mathrm{CO}}_{2}↑+\;20{\;H}_{2}O↑+\;2{\;N}_{2}↑$$

#### 2.1.2. Synthesis of MoS_2_/g-C_3_N_4_

g-C_3_N_4_ was added to MoS_2_ (1:1 *w*/*w*, 300 mg of each), the mixture was dispersed in 30 mL of propylene carbonate and stirred at 40 °C for 30 min, followed by sonication for 30 min in four periods of 5 min each, without exceeding 40 °C. By centrifugation of this solution, a precipitate was obtained, which was dried at 150 °C for 24 h to obtain a MoS_2_/g-C_3_N_4_ composite material similar to those previously reported in the literature [17,18,19].

#### 2.1.3. Synthesis of MoO_3_

Nanostructured *α*-MoO_3_ was obtained by heating 500 mg of the hydrothermally synthesized MoS_2_/g-C_3_N_4_ composite in air at 650 °C for 30 min in a Al_2_O_3_ ceramic crucible with lid in a GVA 12/900 oven (Carbolite Gero, Hope Valley, UK; power: 5.460 kW; heating length: 900 mm; T_max_: 1200 °C). Thermal heating of the composite at 650 °C gave molybdenum trioxide crystals with a multilayer stacked structure. Carbon nitride oxide, (g-C_3_N_4_)O, formed from g-C_3_N_4_, was released as gaseous vapor [20].

(3) $$2{\;\mathrm{Mo}}_{2}S/{\mathrm{g-C}}_{3}N_{4}+9{\;O}_{2}\to 4{\;\mathrm{MoO}}_{3}+2{\;\mathrm{SO}}_{2}↑+\;2\;\left({\mathrm{g-C}}_{3}N_{4}\right)O↑$$

### 2.2. Characterization

The vibrational spectrum in the 400–4000 cm^−1^ spectral range was characterized using a Thermo Scientific (Waltham, MA, USA) Nicolet iS50 Fourier-transform infrared (FT-IR) spectrometer, equipped with an in-built diamond attenuated total reflection (ATR) system, with a 1 cm^−1^ spectral resolution and 64 scans.

The X-ray powder diffraction pattern was obtained with a Bruker (Billerica, MA, USA) D8 Advance powder diffractometer in a Bragg-Brentano geometry, using a silicon crystal low background specimen holder. Data was collected in the 2*θ* = 5°–80° range, with increments of 0.01° and an acquisition time per step of 0.5 s.

Scanning electron microscopy (SEM) analysis was carried out with a Tescan (Brno, Czech Republic) Vega3 microscope with BSE (annular, YAG crystal, 0.1 atomic resolution) and SE (Everhart-Thornley type, YAG crystal) detectors, and equipped with a Bruker Quantax 100 Easy energy-dispersive X-ray analysis (EDX) system based on a Bruker Xflash 410 M Silicon Drift Detector, with a 133 eV energy resolution (Mn Ka) @ 100 kcps. Transmission electron (TEM) micrographs and the selected area electron diffraction (SAED) pattern were obtained in a JEM FS2200 HRP microscope (JEOL, Akishima, Tokyo, Japan) operating at 200 kV.

The X-ray photoelectron spectroscopy (XPS) spectrum was collected using a Kratos AXIS UltraDLD instrument (Kratos Analytical Ltd., Manchester, UK) with a monochromatic Al Kα X-ray source (1486.6 eV). For energy calibration, XPS binding energies were referenced to the C 1s peak at 284.6 eV.

The diffuse optical reflectance spectrum (UV-Vis DR) was obtained in a CARY 500 spectrometer (Agilent, Santa Clara, CA, USA) equipped with an integration sphere. The spectrum was recorded in diffuse reflectance mode and transformed by the instrument software to equivalent absorption Kubelka-Munk (K-M) units. The K-M function was plotted as a function of energy and the bandgap value was calculated through the inflection point of this curve.

## 3. Results and Discussion

### 3.1. Vibrational Characterization

**Figure 1:**
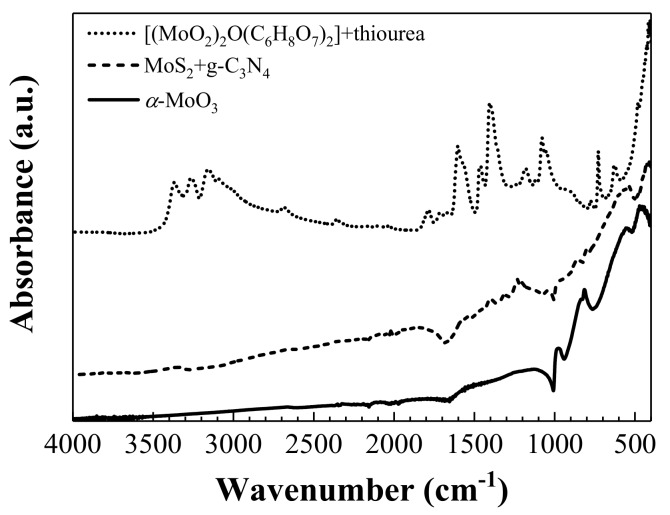
Normalized ATR-FTIR spectra of two intermediate steps of the synthesis and the final *α*-MoO_3_ product. An offset has been added for clarity purposes.

The reactions between molybdate, citrate and thiourea were tracked by ATR-FTIR spectroscopy. As thiourea was added to molybdate-citrate under ambient conditions, a shift towards lower wavenumbers of the C=S stretching peak (from 731 to 728 cm^−1^) and of the C–N stretching peak (from 1473 to 1461 cm^−1^) were observed. These shifts pointed at bond formation between Mo of molybdate and S of thiourea to yield either a molybdenum-thiourea complex or a MoS_2_ chalcogenide. However, changes in shape and position of NH_2_ and C=O stretching peaks denoted a strong interaction between citrate and the molybdenum-thiourea complex (NH_2_ stretching peaks at 2800 and 3700 cm^−1^ were different from those of molybdenum-thiourea and the C=O stretching peak was shifted from 1624 to 1604 cm^−1^) (Figure 1, dotted line). Presence of some MoS_2_, even before treatment in the Parr reactor, could be observed in the peak at 482 cm^−1^, which corresponded to γ_as_ (Mo–S) [21].

Upon addition of g-C_3_N_4_ (Figure 1, dashed line), the spectra showed a band at 1204 cm^−1^ due to C/N networks. The peak at 806 cm^−1^ could be either assigned to heptazine ring, to a bending mode of tris-*s*-triazine or to the Mo_2_–O stretching modes of MoO_3_. The peak at 541 cm^−1^ was due to νCS vibration. Mo–S characteristic vibration was shifted to 475 cm^−1^.

Finally, upon treatment at 650 °C, MoS_2_ was oxidized to MoO_3_ (Figure 1, solid line). The Mo=O vibration was observed at 1126 cm^−1^. The peak at 977 cm^−1^ corresponded with the Mo–O bonds. The peak at 815 cm^−1^ was due to the doubly connected bridge-oxygen Mo_2_–O stretching modes of doubly coordinated oxygen, caused by corner-shared oxygen atoms in common to two MoO_6_ octahedra [22]. The peak at 556 cm^−1^ was characteristic of stretching vibrations of Mo–O. Some remains of MoS_2_ were identified by the Mo–S vibration at 471 cm^−1^. 

### 3.2. X-Ray Powder Diffraction and Energy-Dispersive X-Ray Spectroscopy Analyses

**Figure 2:**
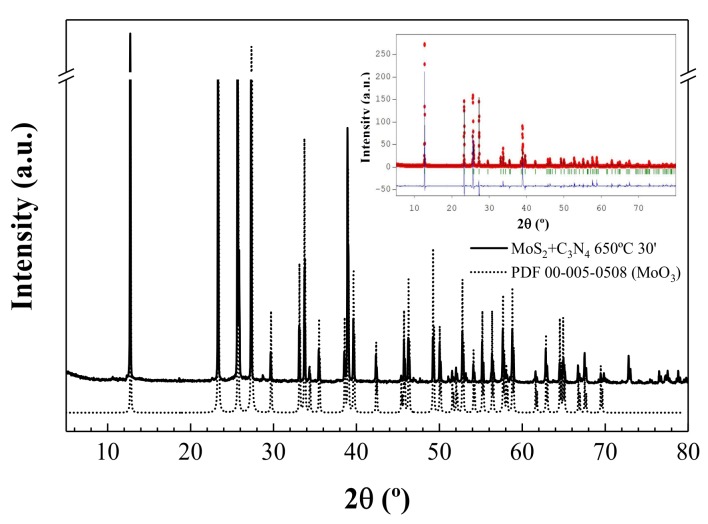
X-ray powder diffraction patterns for the end product upon treatment at 650 °C (solid line) and for orthorhombic *α*-MoO_3_ (dotted line). Inset: Rietveld refinement results, using FullProf [23].

The X-ray powder diffractogram of the end product for a treatment temperature of 650 °C (Figure 2) matched well the one reported in ICDD crystallographic database for orthorhombic *α*-MoO_3_ (PDF 00-005-0508). The positions of the experimental peaks were in good agreement with the simulated diffractogram, albeit with changes in the intensity, which may be explained by preferential orientation resulting from the Bragg-Brentano geometry used in the data collection.

**Figure 3:**
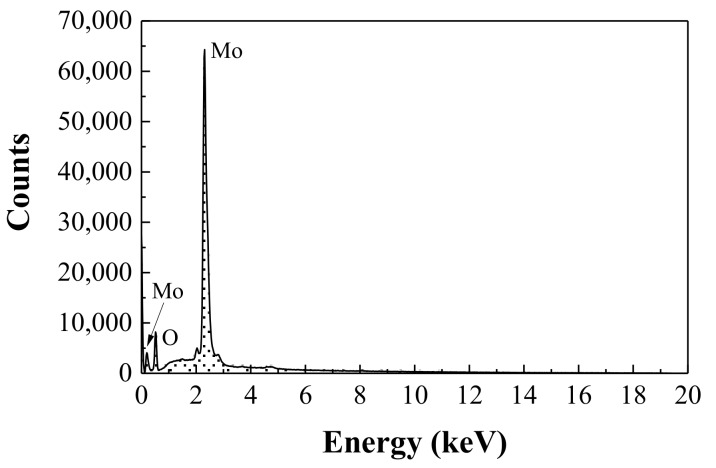
EDX analysis of the end product.

The EDX analysis (Figure 3 and Table 1) resulted in a molybdenum to oxygen atomic ratio *A*_Mo:O_ = 0.29, in reasonably good agreement with the theoretical 0.33 ratio. It also pointed at the presence of aluminum impurities, tentatively ascribed to contamination from the crucible.

### 3.3. Scanning and Transmission Electron Microscopy Analyses

**Figure 4:**
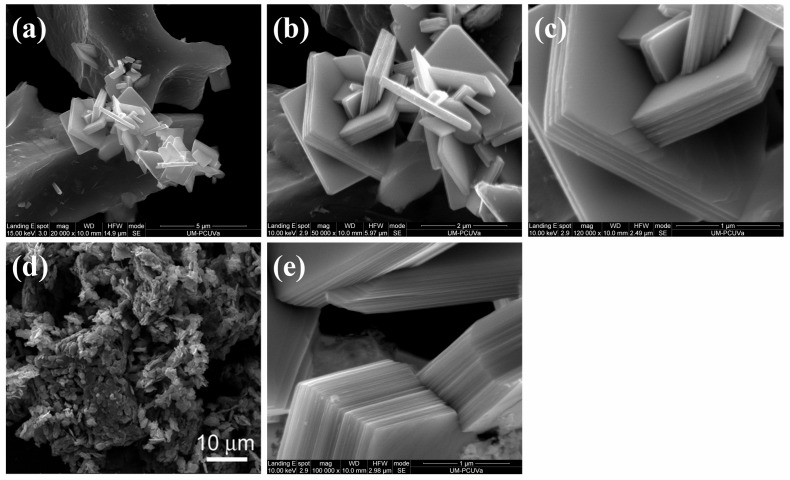
(**a**–**c**) SEM micrographs of *α*-MoO_3_ crystals with a multi-layer stack structure at different magnifications (20,000×, 50,000× and 120,000×, respectively); (**d**) SEM image of MoO_3_ crystals obtained by calcination of commercial molybdic acid (MoO_3_·H_2_O); (**e**) SEM micrograph of *α*-MoO_3_ stacking with 44 layers.

Molybdenum trioxide obtained by the procedure reported above was a greenish-white crystalline material. SEM micrographs revealed a multilayer stack structure built from planar crystals, either 1×1 µm or 2×2 μm in size (Figure 4a–c). The shape of the crystals was similar to those reported by Wang, et al. [24], [25] or Vila, et al. [26], corresponding to *α*-MoO_3_, and was very different from that obtained by calcination of commercial molybdic acid, MoO_3_·H_2_O (Figure 4d).

In the SEM micrographs presented above, the number of stacked layers varied between 3 and 44. For a stacking of 1 µm of thickness (Figure 4e), 44 layers could be discerned. The spacing between layers derived from the cross-section SEM images (25 nm) was around 18 times the thickness of two double-layers within a unit cell of the orthorhombic *α*-MoO_3_ crystal.

**Figure 5:**
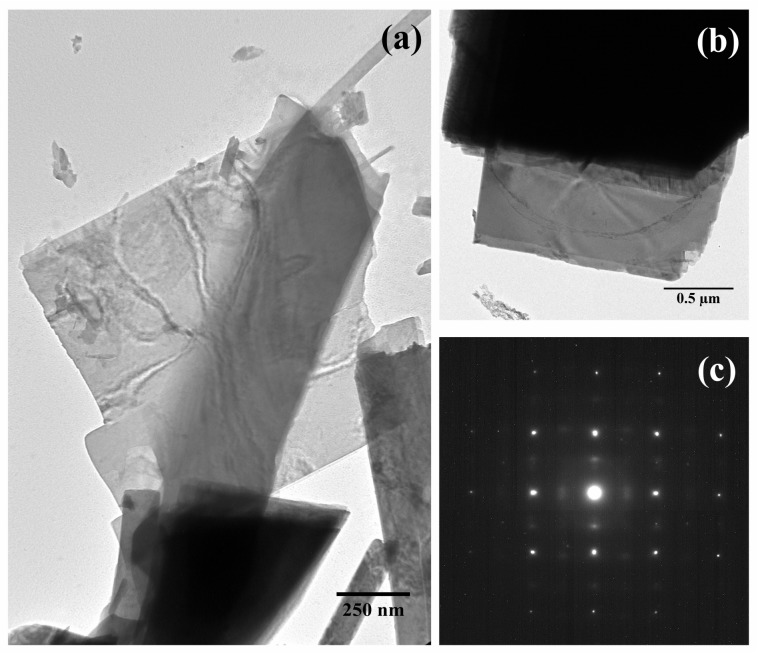
(**a**,**b**) TEM micrographs of the *α*-MoO_3_ nanoplates; (**c**) SAED pattern.

Figure 5a,b shows TEM micrographs of the *α*-MoO_3_ nanoplates, similar to those obtained, for instance, by calcination of h-MoO_3_ microrods [27]. The SAED pattern (Figure 5c) was indexed to correspond with the (002), (202), and (200) crystallographic planes, which were specified as orthorhombic *α*-MoO_3_ [28], in accordance with the XRD analysis.

### 3.4. Surface Characterization

**Figure 6:**
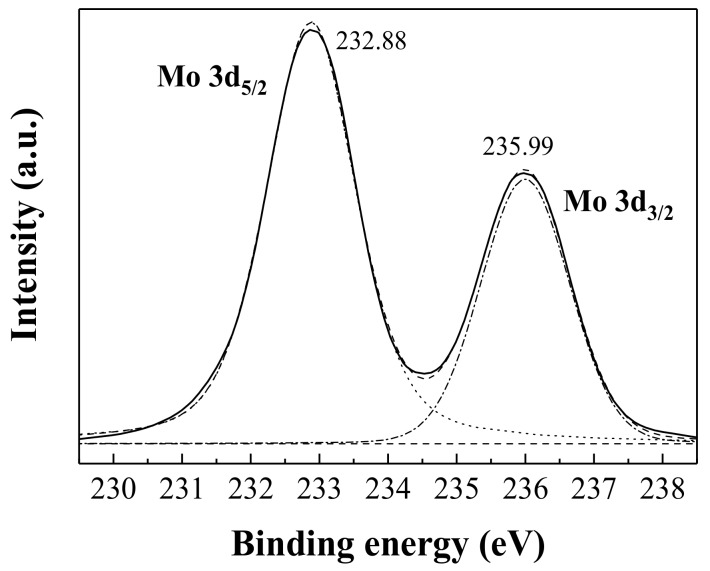
High resolution scan of Mo 3d doublet core levels of *α*-MoO_3_.

Figure 6 shows the Mo (3*d*) XPS spectrum of the *α*-MoO_3_ sample. The doublet at 232.88 and 235.99 eV are attributed to the binding energies of the 3*d*_5/2_ and 3*d*_3/2_ electrons of Mo^6+^, respectively, in good agreement with previous reports of Mo^6+^ state of *α*-MoO_3_ [29,30].

### 3.5. Optical Properties

**Figure 7:**
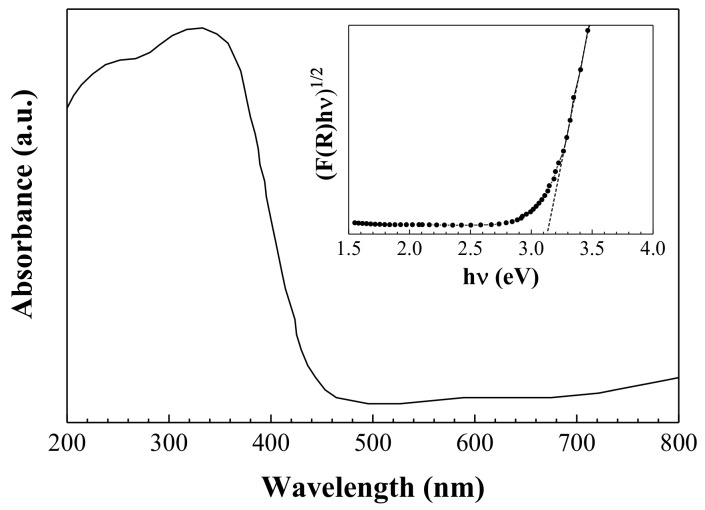
Diffuse reflectance spectrum and band gap energy (inset) of *α*-MoO_3_.

To examine the optical properties of the sample, its UV-Vis DR spectrum was recorded over the 200–800 nm wavelength range at room temperature. As shown in the inset in Figure 7, the optical band gap was found to be ~3.1 eV, which is in good agreement with values reported in the literature [25,31].

## 4. Conclusions

A novel method for the preparation of high quality *α*-MoO_3_ was proposed, based on the use of g-C_3_N_4_ as a lamellar template for the calcination of MoS_2_ (previously obtained from molybdate, citrate and thiourea) at 650 °C. The resulting orthorhombic molybdenum oxide was characterized by X-ray powder diffraction, ATR-FTIR, SEM, TEM, EDX, XPS and UV-Vis DR. X-ray powder diffraction data confirmed the good crystallinity of the obtained product, while the micrographs evinced the presence of well-defined large nanoplates, comparable to those obtained by vapor-phase synthesis techniques. The proposed procedure may thus pose an alternative to PVD and CVD methods, as it can overcome some of their limitations of in terms of energy requirements and equipment, and to conventional liquid-phase synthesis techniques, provided that it can result in higher crystallinity.

## Figures and Tables

**Table 1:** Estimated chemical composition of the end product. Data were obtained with EDX in semi-quantitative mode. The errors were automatically calculated by the analysis software.

Element	Series	[wt.%]	[norm. wt.%]	[norm. at.%]	Error in wt.% (3σ)
Oxygen	K-series	25.473	36.624	77.403	9.409
Aluminum	K-series	0.070	0.100	0.126	0.089
Sulfur *	K-series	0.172	0.247	0.124	0.097
Molybdenum	L-series	43.839	63.029	22.210	4.732

* The percentage assigned to sulfur may be ascribed to limitations of the software in the discrimination of molybdenum and sulfur by peak deconvolution.
